# CRISPR-Cas13a-Based Detection for Bovine Viral Diarrhea Virus

**DOI:** 10.3389/fvets.2021.603919

**Published:** 2021-06-09

**Authors:** Rui Yao, Yueren Xu, Lang Wang, Dawei Wang, Linchang Ren, Changling Ren, Cunyuan Li, Xiaoyue Li, Wei Ni, Yanhua He, Ruirui Hu, Tao Guo, Yaxin Li, Lei Li, Xiaokui Wang, Shengwei Hu

**Affiliations:** ^1^College of Life Sciences, Shihezi University, Shihezi, China; ^2^College of Animal Science and Technology, Shihezi University, Shihezi, China; ^3^State Key Laboratory of Sheep Genetic Improvement and Healthy Production, Xinjiang Academy of Agricultural and Reclamation Sciences, Shihezi, China

**Keywords:** CRISPR-Cas13a, BVDV, detection, virus, LwCas13a

## Abstract

Bovine Viral Diarrhea Virus (BVDV) is the main pathogen of bovine viral diarrhea disease (BVD), which leads to enormous economic losses in the cattle industry. A sensitive and specific detection for BVDV is advantageous to the control of BVDV. Clustered regularly interspaced short palindromic repeats (CRISPR)-Cas systems have been used for detecting virus RNA. In this study, the expression and purification of LwCas13a protein was optimized and the RNase activity of LwCas13a *in vitro* was verified. CRISPR-LwCas13a system could detect BVDV virus and BVDV RNA with high specificity and simplicity. The detection limit of the LwCas13a system was 10^3^ pM, and there were no cross-reactions with HEK293T and MDBK. In summary, a sensitive, specific, and simple nucleic acid detection method based on CRISPR-Cas13a was developed for BVDV. This method provides a new detection strategy for early diagnosis of BVDV.

## Introduction

Bovine Viral Diarrhea Virus (BVDV) is a high-prevalence viral of cattle, affecting herds worldwide and leading to significant economic losses in the cattle industry (1). BVDV is a positive-sense single-standard ribonucleic acid (RNA) virus that belongs to *Pestivirus* genus and *Flaviviridae* family (2). The disease results in multiple clinical symptoms in the respiratory system, digestive tract, and reproductive system (3–5). In addition, recessive infections of BVDV result in consequences of abortion, which is a key feature in the epidemiology of this disease (6). Thus, control options for reducing BVDV infections were based on initial screening for serological evidence of persistently infected animals (7). At present, there are many methods of detection for BVDV virus, including traditional serological neutralization test, immunohistochemical method, ELISA, and, recently, PCR technique (8). Traditional methods of BVDV are costly and need precise instruments (9), so it is very urgent to establish a fast, accurate, efficient, and visual method to detect BVDV.

Recently, CRISPR-associated (CRISPR-Cas) based nucleic acid detection platforms (10) with high sensitivity and specificity have been developed. Shnmakov et al. (11) identified 53 candidate genes, which can be divided into three categories, namely C2C1, C2C2, and C2C3. In 2016, Cas13a (before known as C2C2) was shown to be able to detect the presence of RNA target by CRISPR RNA (crRNA) and collateral cleavage activity of Cas13a (12). It contains two HEPN domains with RNase activity, responsible for the processing maturation of crRNA and the degradation of target RNA, respectively (13–17). Subsequently, *in vitro* experiments found that, after complementary pairing of crRNA and target single-stranded RNA, Cas13a was combined to form a complex and underwent a conformation change, activating non-specific RNase activity that could target cleavable accessible single-stranded RNA (ssRNA) (18–20). Among them, Gootenberg found that *Leptotrichia wadei* Cas13a (LwCas13a) that activated non-specific RNase activity could detect Zika and Dengue viruses in samples (19). Similarly, the SHERLOCK platform can use the Cas13 enzyme's RNA cleavage activity to achieve the detection of RNA viruses, which provides a new detection strategy for RNA viruses such as BVDV (20). These studies show that the detection method based on CRISPR-Cas13a has the characteristics of high sensitivity and specificity for the detection of diseases.

In this study, our aim is to develop a CRISPR-Cas13a-based diagnostic technology system for BVDV detection. A pair of specific crRNAs was designed based on the reported BVDV gene sequence in the 5'UTR conserved region, and Reporter (quencher fluorescent RNA) was synthesized. The expression and purification of LwCas13a protein were optimized, and the RNase activity of LwCas13a *in vitro* was measured. The sensitivity and specificity of the LwCas13a-based detection system was evaluated. A new nucleic acid detection method for the BVDV virus has been developed, which provides a new strategy for field detection of BVDV and an efficient and practical platform for RNA virus detection.

## Materials and Methods

### Experimental Materials

The Twinstrep-SUMO-huLCas13a plasmid was generously donated by Zhang Feng Laboratory (Broad Institute, USA). The MDBK cell line and HEK-293T cell line were purchased from the Chinese Academy of Sciences. The BVDV (NADL strain, GenBank accession number: M31182) was preserved in our laboratory.

### Primer Design

The full length of pC013-twinjstrep-sumo-LwCas13a gene was obtained on addgene (http://www.addgene.org/). Design of LwCas13a Protein Full-length Primers was carried out with Premier 5 (Premier Biosoft International, San Francisco, CA, USA). The primers were synthesized by Beijing Ruiboxingke Biotechnology Co., Ltd. (Table 1). The BVDV (NADL strain) specific identification primers were selected in the conserved region of the gene sequence and synthesized by Shanghai Sangon Biological Engineering Technology Service Co., Ltd. (Table 1).

**Table 1:** Sequence of primers, crRNA, and Reporter (quencher fluorescent RNA).

**Name**	**Sequence (5^**′**^-3^**′**^)**	**References**
Lwa13a-F	CATGCGGGCAGGAAGCAAGGACGACGTCG	This study
Lwa13a-R	CGGGAGCCCCCTCTCTCTCTCTCTCTCTCTTTGTACGA	
BVDV-F	ATGCCCTTAGTAGGACTAGCA	This study
BVDV-R	AACGCTTCACGAATTTGCGT	
CrRNA	GGGGAUUUAGACUACCCCAAAAACGAAG	This study
	GGGACUAAAACGCCAUCCAACGAACUCA	
	CCACUGUUGCU	
Reporter	6-FAM/GAAGAGAGUUUUAUUCAGAUAGA	(17)
	UUUGU/BHQ-1	

CRISPR-RNA (crRNA) preparation, the design of crRNA was carried out according to NADL strain select BVDV sequence that contains the highly conserved sites of the 5'UTR region, and these were then were synthesized by TaKaRa (Dalian) Co., Ltd. (Table 1). The fluorescence (FAM)-and quencher (BHQ1)-labeled RNA Reporter (FQ5U) is RNA with 5′ modified to FAM, 3′ modified to BHQ-1 (Table 1).

### Expression and Purification of LwCas13a Protein

LwCas13a protein purification was performed as previously described with some modifications (17). The full-length gene sequence of LwCas13a (3,491 bp) was synthesized by PCR and cloned into a prokaryotic expression vector pET-28a^+^ (ThermoFisher Scientific, Shanghai, China). And then, the LwCas13a recombinant Plasmid was transformed into Rosetta™ (DE3) pLysS Singles Competent Cells (TransGen Biotech, Beijing, China). Sixteen milliliters overnight starter culture was added into Terrific Broth 4 L growth media (12 g/L tryptone, 24 g/L yeast extract, 9.4 g/L K_2_HPO_4_, 2.2 g/L KH_2_PO_4_, Sigma) (TB). Subsequently, LwCas13a was inoculated at 37°C and 200 rpm until an OD_600_ of 0.6. Meanwhile, the culture was supplemented with 500 uM (final concentration) IPTG (Isopropyl-1-thio-d-galactopyranoside) and induced for protein expression at 18°C for 20 h.

The culture was centrifuged at 8,000 rpm for 15 min at 4°C and the cell pellet was then collected. The cell pellet was then crushed and suspended in lysis buffer (20 mM Tris-HCL, 300 mM NaCl, 1 mM DTT, pH 8.0), and then supplemented with Lysozyme (Sigma), benzonasefor® Nuclease, and protease inhibitors (Complete Ulter EDTA-free tablets) followed by sonication (Ningbo Xinzhi Scienz-IID) under the following conditions: power 60%, 2 s on and 3 s off, the total sonication time is at least 30 min until the lysate cleared. It was then centrifuged for 30 min at 4°C at 12,000 g and the supernatant was harvested.

The supernatant was filtered through 0.22 uM and applied to 5 mL HisTrapTM crud FF. Then, the bound protein was eluted with buffer (20 mM NaH_2_PO_4_, 500 mM NaCl, 500 mM imidazole pH 7.4) according to the operation method as specified in the manual (GE Healthcare Life Sciences). This was confirmed by SDS-PAGE and Coomassie blue staining. After, the elution buffer was concentrated on protein storage buffer (600 mM NaCl, 50 mM Tris-HCl pH 7.5, 5% glycerol, 2 mM DTT) by centrifugation filter unit (Millipore ultrafiltration centrifuge tube). The LwCas13a protein was stored at −80°C.

### Cell Culture and Virus RNA Extraction

MDBK cells and HEK293T cells were cultured in high-glucose Dulbecco's modified Eagle's medium (DMEM; Hyclone, USA) supplemented with 10% fetal bovine serum (FBS) (Hyclone, USA) (GM) at 37°C with 5% CO_2_ for constant temperature culture, and then the cell was collected. MDBK cells in the logarithmic phase were used in the experiment; after growing to ~80% confluency, the fused virus suspension of BVDV (NADL strain) was added into the cell culture medium and allowed to culture until more than 80% of the cells' pathological changes. Finally, virus solution was collected using Trizol (Thermo Fisher Scientific) and was stored at −80°C until use.

The viral RNA of BVDV was extracted using QIAamp Viral RNA Mini Kit (QIAGEN, Duesseldorf, Germany) according to the manufacturer's instruction. Subsequently, total RNA was reverse transcribed into cDNA using the Prime Script RT Master Mix (Takara, Dalian, China) according to the manufacturer's instruction. MDBK cells and HEK293T cells were used to extract the RNA. All RNA and cDNA were stored at −20°C until use.

### CRISPR-Cas13a Detection System

**Figure 1:**
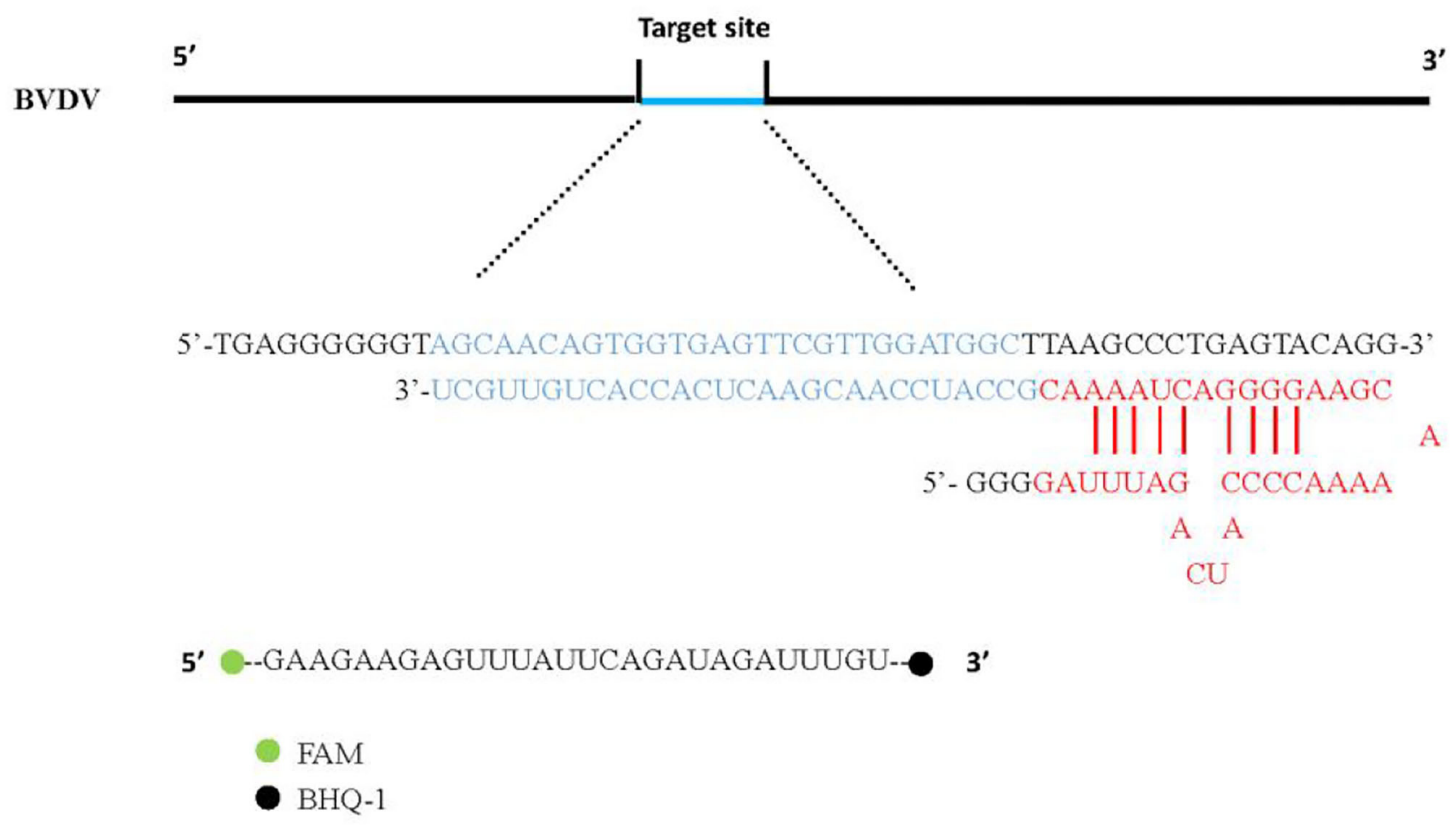
CrRNA and Reporter pattern model. The hairpin structure at the 5' end of crRNA was recognized by the REC (crRNA recognition) lobe of Cas13a protein. Cas13a produces RNase activity in the presence of target RNA and releases fluorescent signals.

We first verify the activity of the LwCas13a protein. The biochemical characterization of LwCas13a revealed crRNA mediated RNA cleavage promoted by two HEPN nuclease domains. Upon recognition of its RNA target, crRNA and Cas13a can bind to complement and trigger LwCas13a, which engages in “collateral” cleavage of nearby non-targeted RNAs. As a result, the fluorescence (FAM)-and quencher (BHQ-1) -labeled RNA probe in Figure 1 as the reporter probe is digested by LwCas13a. Thus, it can be indicated that the complementary pairing of crRNA and the target single-stranded RNA leads to LwCas13a binding to form a complex to exert RNA cleavage activity.

The detection system included: 45 nM purified LwCas13a, 22.5 nM crRNA, 125 nM RNA Reporter (Takara, Dalian, China), 2 uL murine RNase inhibitor (New England Biolabs), 100 ng of background RNA (purified from HEK293T), varying amounts of BVDV RNA, and LwCas13a detection buffer (40 mM Tris-HCl, 60 mM NaCl, 6 mM MgCl_2_, pH 7.3). The total reaction volume was 40 uL. The mixture was incubated in a 96-well plate on Synergy HTX Multi-Mode Reader (BioTek, a part of Agilent) at 37°C for 1 h with fluorescence kinetics measurements (FAM channel, λex 485 nm, λex 528 nm) taken every 5 min.

### Quantitative PCR Test

According to the manufacturer's instructions, the total RNA in the BVDV was extracted using QIAamp Viral RNA Mini Kit (QIAGEN, Duesseldorf, Germany). The directly extracted RNA cannot be detected with sensitivity due to partial degradation or loss during the process and because it contains multiple RNA fragments. Therefore, we used real-time fluorescence quantitative PCR (RT-qPCR) to make a set of standard curves for the standard RNA, and used the standard curve to quantify the virus sample RNA. Subsequently, the expressed RNA standard was diluted at a 10-fold ratio, and five diluted components were selected as the standard for RT-qPCR. All the samples of each condition were three biological replicates. The RNA concentration of the obtained BVDV virus sample was divided into eight groups by a 10-fold dilution method for the determination of the sensitivity of the CRISPR-Cas13a system.

### Statistical Analysis

All data in this study was analyzed using the one-way analysis of variance (ANOVA) and GraphPad Prism 8 software (Harvey Motulsky, GraphPad Software) for drawing analysis. The pictures in the article were processed by Photoshop CS6 (Charles Geschke, Adobe).

## Results

### Expression and Purification of LwCas13a Protein

**Figure 2:**
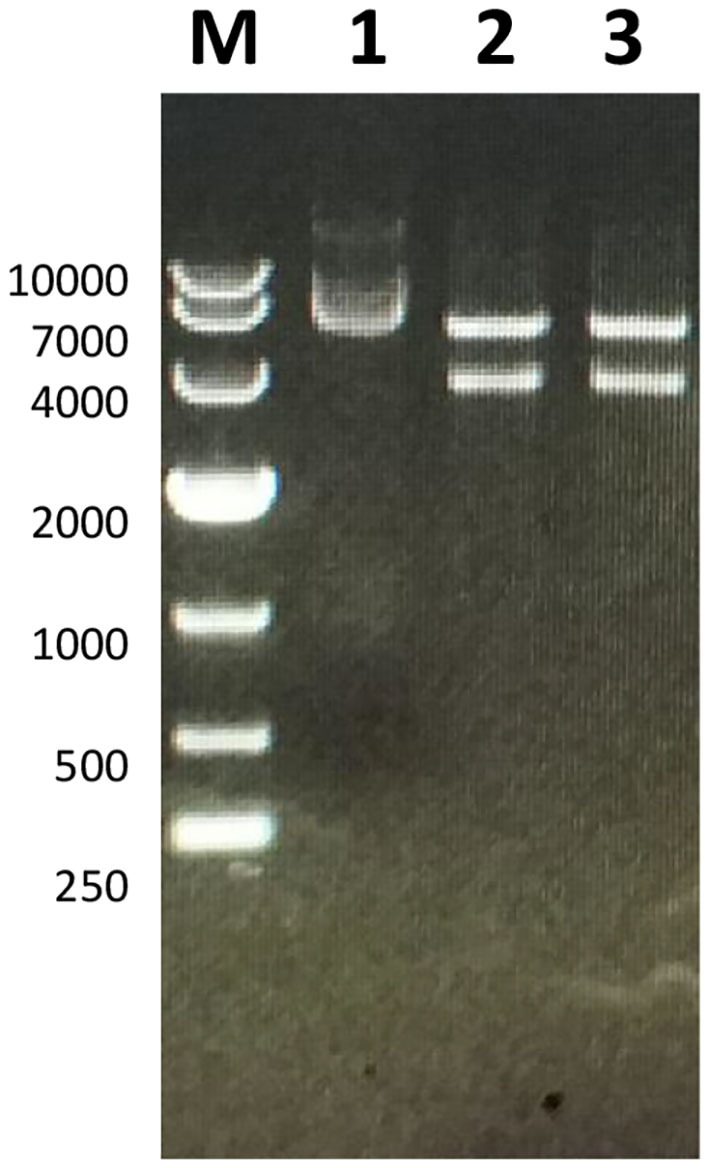
Electropherogram of Twinstrep-SUMO-hu LwCas13a plasmid. M: DL 10,000 DNA Marker; 1: Plasmid extracted from *E. coli*; 2,3: Double-digested plasmid.

**Figure 3:**
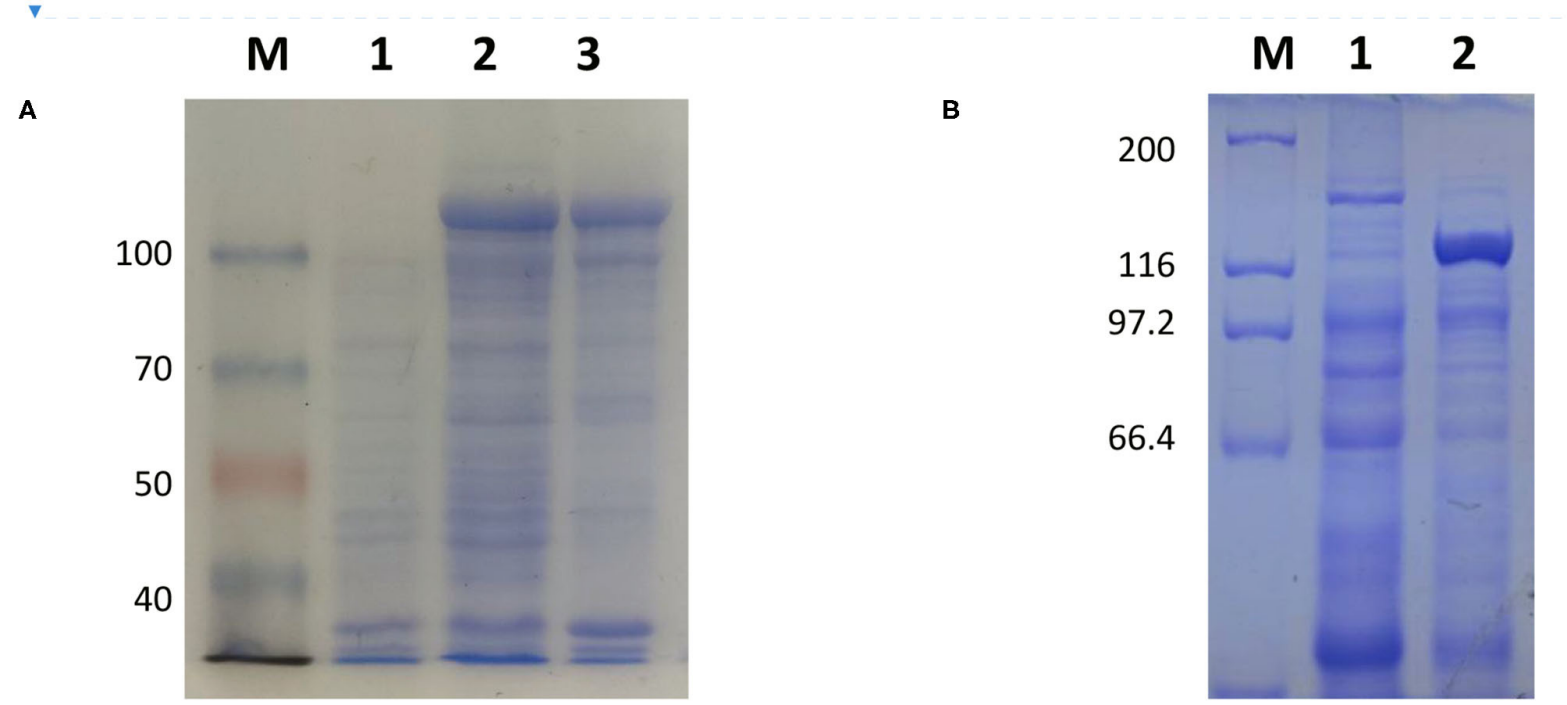
Coomassie blue staining of LwCas13a protein. **(A,B)** Inducible expression of LwCas13a protein at 18°C using 500 uM IPTG. Supernatant of lysed cells was analyzed by SDS-PAGE and stained by Coomassie blue. **(A)** M: 100 kDa Priten marker; 1: Uninduced sample 2: Induce sample supernatant, 3: Induced sample of precipitation Purified. **(B)** M: 200 kDa Priten marker; 1:Uninduced sample, 2: purification of LwaCas13a protein.

The full-length sequence of LwCas13a (3,491 bp) was amplified by PCR and cloned into the pET-28a^+^ vector. As shown in Figure 2, recombinant plasmid pET-28a^+^-LwCas13a could be digested into two fragments. The resulting fragment had an expected size and was verified by DNA sequencing. Then, the recombinant plasmid was transformed into Rosetta™ (DE3) for analysis of the LwCas13a protein expression. As shown in Figure 3, the LwCas13a protein was expressed mainly in the supernatant with 138.5 kD. The purified LwCas13a protein was concentrated and then quantified using BCA protein assay kit (Abcam, Shanghai, China).

### Validation of the CRISPR-Cas13a Detection System

**Figure 4:**
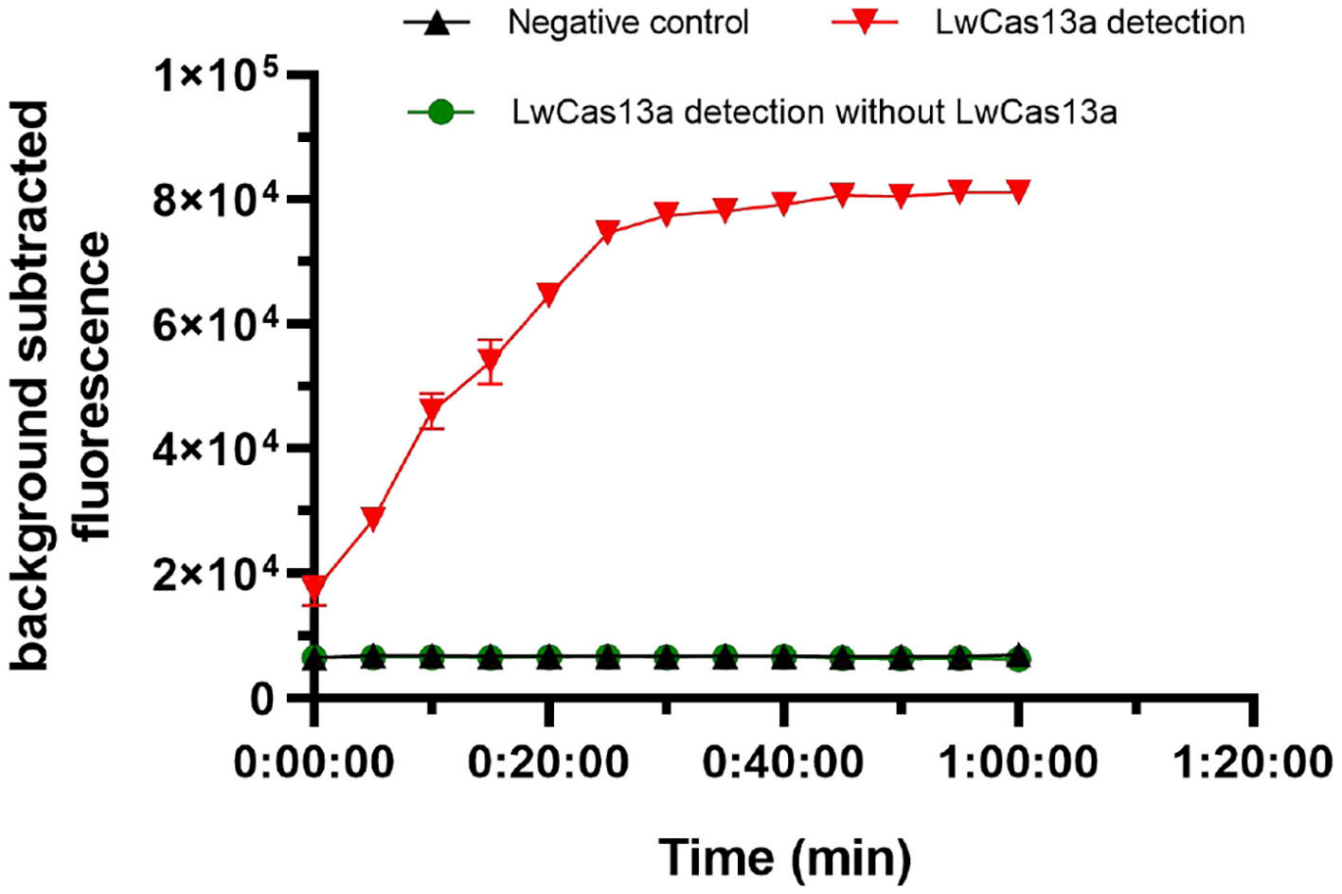
Activity of purified LwCas13a protein (*n* = 3 technical replicates; values represent mean ± SEM).

We next tested the activity of the expressed LwCas13a protein, and the complete LwCas13a reaction system were assessed. As shown in Figure 4, the background subtracted fluorescence of the complete LwCas13a reaction system increased rapidly with time until the peak value, and there was no reaction in the LwCas13a reaction system without LwCas13a protein. The results showed that the crRNA sequence recognition of RNA target was critical for engaging “collateral” cleavage of nearby non-targeted RNAs. The reporter probe was digested by LwCas13a, thus releasing substantial fluorescence signals.

### Specificity of the CRISPR-Cas13a Detection System

**Figure 5:**
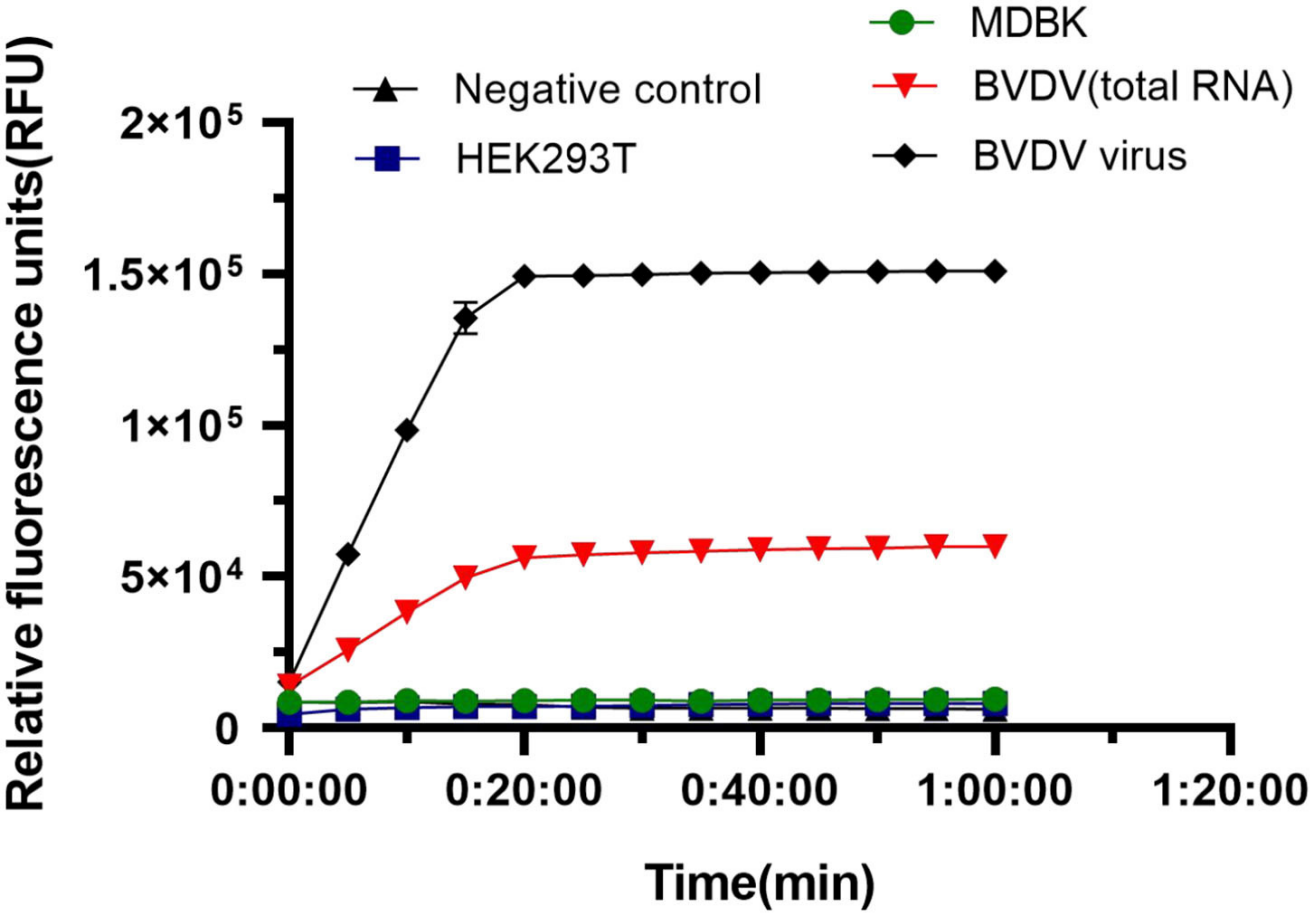
Specificity of LwaCas13a detection. Specificity of LwCas13a fluorescence detection (*n* = 3 technical replicates; values represent mean ± SEM).

We tested the specificity of the LwCas13a system for BVDV detection. We used HEK293T, MDBK, and BVDV virus as controls to detect specificity. The rapid reaction against BVDV virus and BVDV RNA occurred, and there was no cross-reaction with controls (Figure 5). The results showed that the fluorescence intensity of BVDV virus and BVDV RNA detection was significantly higher than that of HEK293T cells and MDBK cells and blank controls. The crRNA used to identify BVDV viral RNA has better specificity than the viral solution. The system can be used to specifically detect the BVDV virus.

### Sensitivity of the CRISPR-Cas13a Detection System

**Figure 6:**
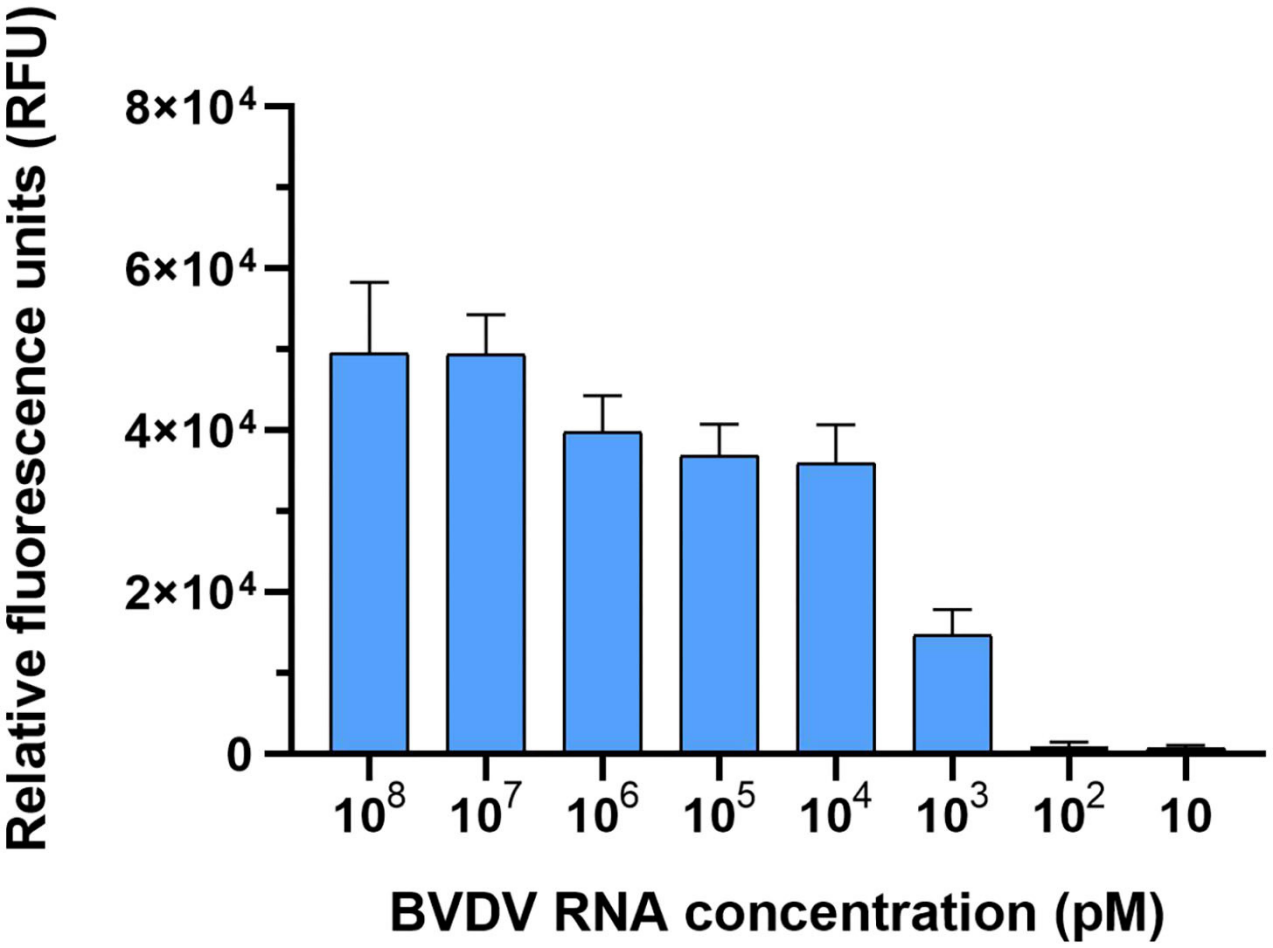
Sensitivity of LwaCas13a detection. Sensitivity of LwCas13a fluorescence detection (*n* = 3 technical replicates; values represent mean ± SEM).

We tested the detection sensitivity of the LwCas13a detection system. The sensitivity of the LwCas13a detection system was tested with 10-fold serial diluted template BVDV (NADL). As shown in Figure 6, six orders of magnitude from 10^8^ pM down to 10^3^ pM template could be detected. These data showed that the detection limit of the LwCas13a system was 10^3^ pM.

## Discussion

Bovine Viral Diarrhea Virus (BVDV) is an economically significant viral disease in the global cattle industry. Since the first identification in China in 1980 (21), BVDV has spread widely throughout the country, making the prevention and control of BVDV more difficult. High variability and recombination among different BVDV strains makes the prevention and control of BVDV more complicated, leading to tremendous economic losses in the cattle industry (22). The CRISPR-Cas13a has had a significant impact in probing molecular detection based on genome editing and is now promising to provide new strategies in the development detection of RNA viruses. Our experiment has explored a new detection method for BVDV, which verifies the feasibility of CRISPR-Cas13a-based detection of NADL strain. Detection of other subtypes will be tested in future experiments. Our test method only needs to obtain the RNA sample of the suspected animal, add it to the reaction system, and perform fluorescence detection. The fastest results can be obtained in 1 h. This method can detect the BVDV virus at the gene level.

Sequence analysis revealed that LwCas13a lacks an identifiable DNase catalytic site, and two HEPN domains containing highly conserved R-X-H motif were identified (16). Further functional studies revealed that Cas13a is an RNA-guided RNA-targeting CRISPR effector (19). The recent biochemical analysis found that Cas13a possesses the second RNase activity, the function of which is closely related to crRNA maturation (16). These two RNase activities of Cas13a are mechanistically distinct from each other, responsible for the processing maturation of crRNA and the degradation of target RNA, respectively. It is used to understand how Cas13a recognizes its crRNA and target RNA and elucidate the molecular mechanism of crRNA precursor processing, as well as crRNA-mediated single-stranded RNA cleavage, and determine the feasibility of using Cas13a to detect RNA viruses (23).

Zhang Feng's research is based on LwCas13a molecular detection platform (SHERLOCK), which can distinguish between Zika virus and Dengue virus at low concentrations (17). Although the sensitivity of our detection system of BVDV detection cannot reach pM level. due to the sample of BVDV, we only need to extract the obtained sample's RNA to directly perform the detection. At present, our system is under further study for detecting animal serum samples directly. In addition, the SHERLOCK reagents can not only be freeze-dried for the cold chain. They can also be freeze-dried for long-term storage or can be easily reconstituted on paper for field applications. The method can also be tested at room temperature and away from the laboratory. This is the work that we need to further develop in the future and are currently focusing on.

Increasing evidence has indicated that combining Csm6 with LwCas13a detection can improve the stability of the test and reduce the possibility of false positive readings (18). Further, other studies have shown that LwCas13a collateral activity combined with lateral flow readings can disrupt the FAM-biotin reporter gene, and the results can be detected on commercial horizontal flow bars, thereby achieving visualization. These studies demonstrated that Cas13b from (PspCas13b) can use the RNA-edited programmable A to I Replacement (REPAIR) system to correct mutations in genetic diseases, and can achieve better editing efficiency (24). With the continuous development of detection technology, CRISPR-Cas13a detection method has been applied for many fields, such as diseases and cancer (25). Therefore, further research on CRISPR-Cas will facilitate the detection of other types of RNA virus.

## Conclusions

We have established a method for detecting the NADL strain based on CRISPR-Cas13a. Although the method needs to be constantly improved and strengthened for visualization and field testing, it opens up a new strategy for rapid, stable, and sensitive detection of RNA viruses such as BVDV.

## Data Availability Statement

The original contributions generated for the study are included in the article/supplementary material, further inquiries can be directed to the corresponding author/s.

## Ethics Statement

All experimental procedures in this study were carried out in accordance with the requirements of the Statute on the Administration of Laboratory Animals and was approved by the Institutional Animal Care and Use Committee at Shihezi University (SU-ACUC-08032).

## Author Contributions

RY, YX, LW, DW, WN, and SH conceived and designed the experiment and analyzed and interpreted the data. RY, YX, LR, CL, WN, and SH wrote the manuscript. RY, YX, CL, WN, SH, XL, RH, TG, YL, LL, and XW took part in the expression and purification of LwCas13a protein. RY, YX, and LW participated in RNA extraction and the construction of CRISPR-Cas13a detection system and processed the data. All authors read and approved the final manuscript.

## Conflict of Interest

The authors declare that the research was conducted in the absence of any commercial or financial relationships that could be construed as a potential conflict of interest.
